# Tentorial venous anatomy of mice and humans

**DOI:** 10.1172/jci.insight.151222

**Published:** 2021-11-08

**Authors:** Pashayar P. Lookian, Vikram Chandrashekhar, Anthony Cappadona, Jean-Paul Bryant, Vibhu Chandrashekhar, Jessa M. Tunacao, Danielle R. Donahue, Jeeva P. Munasinghe, James G. Smirniotopoulos, John D. Heiss, Zhengping Zhuang, Jared S. Rosenblum

**Affiliations:** 1 Neuro-Oncology Branch, National Cancer Institute, and; 2 Surgical Neurology Branch, National Institute of Neurological Disorders and Stroke, NIH, Bethesda, Maryland, USA.; 3 Johns Hopkins University, Baltimore, Maryland, USA.; 4 NeuroSimplicity LLC, North Bethesda, Maryland, USA.; 5 Stony Brook University, Stony Brook, New York, USA.; 6 Mouse Imaging Facility, National Institute of Neurological Disorders and Stroke, NIH, Bethesda, Maryland, USA.; 7 Radiology, George Washington University, Washington, DC, USA.; 8 National Library of Medicine, MedPix, Maryland, USA.

**Keywords:** Development, Vascular Biology, Bone development, Brain cancer, Neuroimaging

## Abstract

We recently described a transtentorial venous system (TTVS), which to our knowledge was previously unknown, connecting venous drainage throughout the brain in humans. Prior to this finding, it was believed that the embryologic tentorial plexus regresses, resulting in a largely avascular tentorium. Our finding contradicted this understanding and necessitated further investigation into the development of the TTVS. Herein, we sought to investigate mice as a model to study the development of this system. First, using vascular casting and ex vivo micro-CT, we demonstrated that this TTVS is conserved in adult mice. Next, using high-resolution MRI, we identified the primitive tentorial venous plexus in the murine embryo at day 14.5. We also found that, at this embryologic stage, the tentorial plexus drains the choroid plexus. Finally, using vascular casting and micro-CT, we found that the TTVS is the dominant venous drainage in the early postnatal period (P8). Herein, we demonstrated that the TTVS is conserved between mice and humans, and we present a longitudinal study of its development. In addition, our findings establish mice as a translational model for further study of this system and its relationship to intracranial physiology.

## Graphical abstract

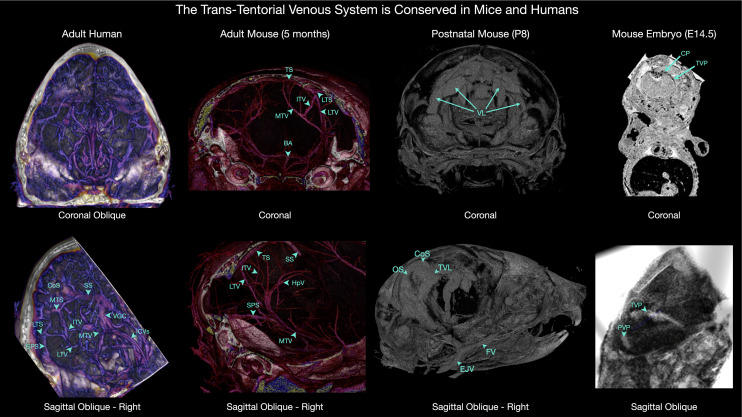

## Introduction

We previously identified a consistent transtentorial venous system (TTVS) in humans that connects venous drainage throughout the brain and proposed its relationship to neurosurgical outcomes, including postoperative remote cerebellar infarct (1, 2). These often-fatal clinical outcomes highlight the need to understand the complete intracranial vascular network. Classically, there are 2 venous drainage systems of the brain, superficial and deep, that develop from the 3 embryologic dorsal venous plexuses, namely the anterior, middle, and posterior (3–5). As dural cleavage forms the tentorium, it is believed that the middle, or tentorial, plexus regresses while the anterior and posterior plexuses coalesce into the superficial venous system and confluence of sinuses, respectively (3, 4). Previous adult human cadaveric studies of the tentorium identified medial and lateral tentorial sinuses (MTS and LTS, respectively), which are outpouchings of the transverse sinuses into the margin of the tentorium that may receive parenchymal bridging veins (6–8). However, these sinuses do not traverse the tentorium to connect distant regions of the brain. Our recent discovery of the TTVS warrants reevaluation of our understanding of the development of the intracranial venous system.

We hypothesized that this TTVS, which we described in a large study of healthy human adults, is the adult derivative of the embryologic tentorial plexus (1, 2). We found this system in humans to be composed of 3 dominant veins with plexiform anastomoses as follows. The medial tentorial vein (MTV) originates from the straight sinus coursing through the free edge of the tentorium to drain into the cavernous sinus (CS). The intermediate tentorial vein (ITV) begins in the straight sinus and can either anastomose with the MTV or continue to the superior petrosal sinus (SPS). The lateral tentorial vein (LTV) has a variable origin and configuration depending on the presence and location of a ring configuration of the MTS and LTS. Terminally, the LTV drains into the SPS. Based on these findings, we described 2 main groups of tentorial venous drainage in humans (1, 2).

We previously suggested that the development of the TTVS is related to cranial development, which in humans continues into the third decade of life (1, 2). Previous studies of human development using spontaneously aborted human fetuses identified tentorial venous drainage of the midbrain at a stage when the developing petrous pyramid narrows the jugular foramen, causing dilation of the transverse sinus (TS) and sigmoid sinus (SS) (8). This dilation leads to outpouching of these structures into the tentorium, which we believe may also lead to the development of the MTS and LTS. To our knowledge, our study was the first to report a consistent TTVS in adult humans (1, 2). In contrast to what has previously been reported, we believe tentorial venous drainage persists, rather than regresses, when jugular venous drainage becomes dominant.

Herein, we combined vascular casting with high-resolution ex vivo imaging, including micro-CT and MRI, to noninvasively investigate the in situ intracranial vasculature in mice. This method allowed us to identify a TTVS in adult mice that is homologous to what we previously described in humans. Further, we studied the development of this system at murine E14.5 and into the early postnatal period (P8) as cranial development continues, which reflects human development (9, 10).

## Results

**Figure 1:**
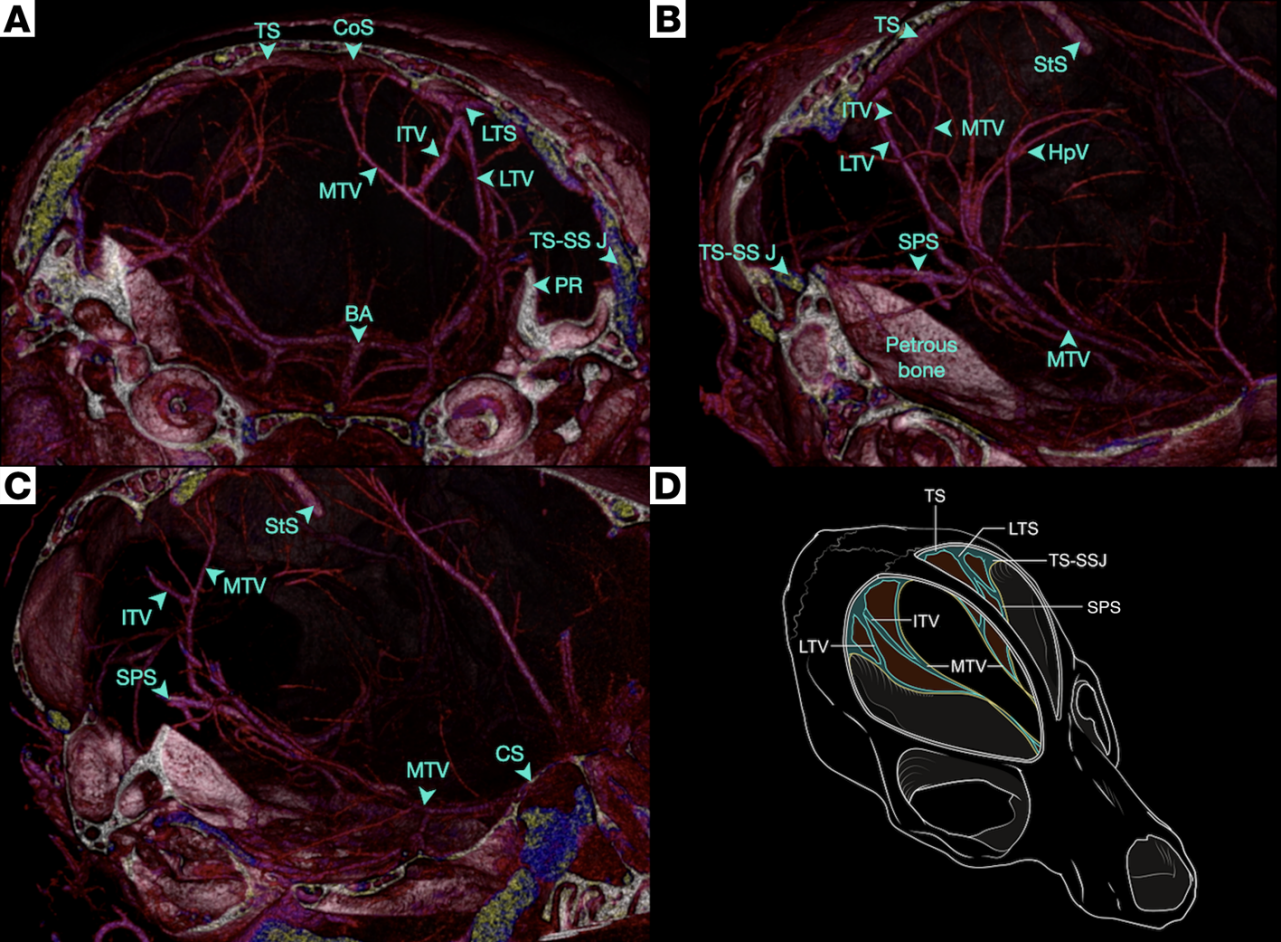
Transtentorial venous system in the adult mouse. (**A**–**C**) A 3D visualization of a representative polymer-perfused sample imaged with micro-CT. (**A**) The annotated TTVS in a coronal view highlighting all vessels within the tentorium. (**B** and **C**) The annotated TTVS in a parasagittal view. The connections of the TTVS with the transverse sinus (TS) and superior petrosal sinus (SPS) posteriorly and the cavernous sinus (CS) anteriorly are highlighted. (**D**) A schematic representation of the TTVS (*n* = 4, 1 male and 3 female). BA, basilar artery; HpV, hippocampal veins; ITV, intermediate tentorial vein; LTS, lateral tentorial sinus; LTV, lateral tentorial vein; MTV, medial tentorial vein; StS, straight sinus; TS, transverse sinus; PR, petrous ridge; TS-SS J, transverse sinus-sigmoid sinus junction; SPS, superior petrosal sinus.

Figure 1 shows the TTVS in situ in adult mice via vascular casting with low-density polymer and micro-CT. We found that the murine TTVS connects distant regions of the brain and has 3 main veins — medial, intermediate, and lateral — with plexiform anastomoses. The MTV runs within the free edge of the tentorium connecting the TS to the CS. The LTS originates from the TS and gives rise to both the LTV and ITV, which continue to anastomose within the tentorium. The SPS, which originates from the TS-SS junction, and MTV connect to the CS along the skull base. Representative 2D raw data and 3D flythroughs are shown in Supplemental Figure 1 and Supplemental Video 1, respectively (supplemental material available online with this article; https://doi.org/10.1172/jci.insight.151222DS1).

**Figure 2:**
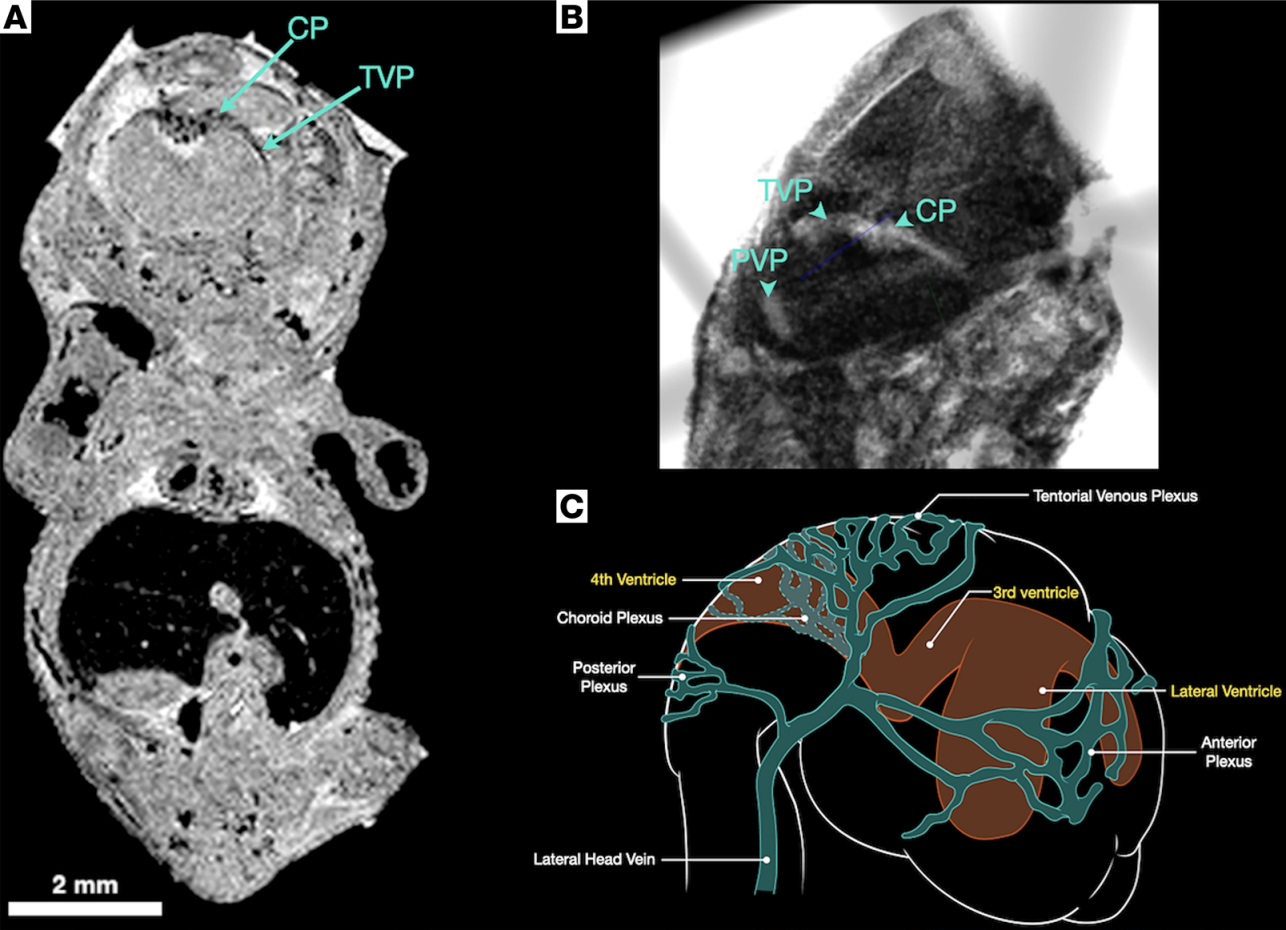
Tentorial venous plexus in the E14. 5 embryo. (**A** and **B**) 2D raw data and 3D visualization of a representative contrast-enhanced embryo imaged with MRI. (**A**) An annotated coronal slice of the embryologic choroid plexus (CP) and tentorial venous plexus (TVP). (**B**) A color-inverted 3D volume render of the same sample with the posterior and tentorial venous plexuses annotated. (**C**) An annotated schematic diagram of the embryologic plexuses and ventricles. (*n* = 4, 2 male and 2 female). Scale bar: 2 mm. PVP, posterior venous plexus.

Figure 2 shows high-resolution ex vivo MRI of the murine embryo at E14.5. At this stage, we found the cranial anterior, middle, and posterior venous plexuses. We found that the middle, or tentorial, venous plexus courses along the superior margin of the developing rhombencephalon to drain into the lateral head vein. We also found that the tentorial venous plexus drains the choroid plexus of the fourth ventricle at this stage. Representative 2D raw data and 3D flythroughs are shown in Supplemental Video 2.

**Figure 3:**
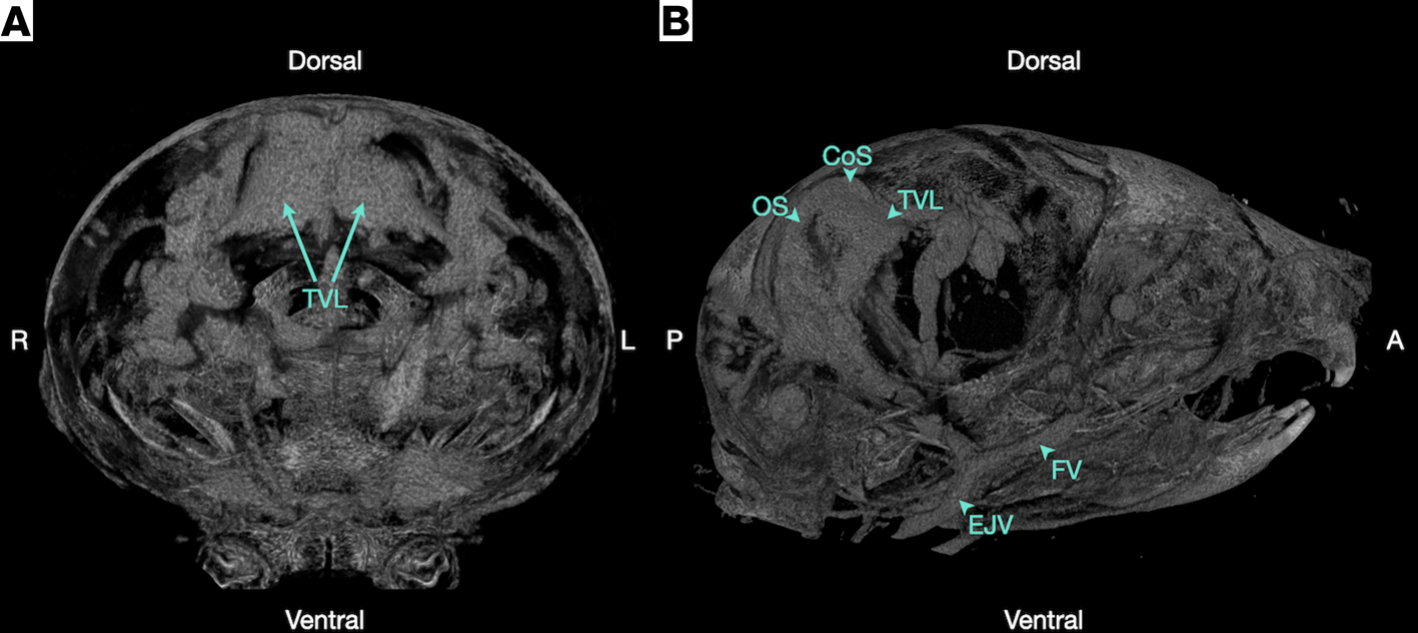
Tentorial venous lakes in the P8 mouse. (**A** and **B**) A 3D visualization of a representative polymer-perfused sample imaged with micro-CT. (**A**) A coronal view of the sample with tentorial venous lakes (TVL) annotated. (**B**) A parasagittal view with large extracranial veins, intracranial tentorial venous lakes, and relevant sinuses annotated (*n* = 3, 1 male and 2 female). CoS, confluence of sinuses; EJV, external jugular vein; FV, facial vein; OS, occipital sinus; R, right; L, left; A, anterior; P, posterior.

Figure 3 shows the intracranial vasculature at P8 after vascular casting with low-density polymer and micro-CT. At this stage, we found that the large intra- and extracranial vessels approach their adult configuration; however, the superior sagittal sinus appears to be absent. The TTVS, which at this stage is an amalgamation of venous lakes with plexiform connections, appears to be the dominant intracranial venous drainage. Representative 2D raw data and 3D flythroughs are shown in Supplemental Figure 2 and Supplemental Video 3, respectively.

## Discussion

Herein, we identified the murine homolog to the TTVS that we previously characterized in 238 healthy adult humans via high-resolution, contrast-enhanced CT (1, 2). Further, in this study, we demonstrated how the embryologic dorsal tentorial venous plexus transitions to this adult configuration in the postnatal period. Our findings in these studies suggest that these veins, previously thought to obliterate during tentorial development based on previous human developmental studies, remain patent in the adult as continuous venous drainage between the anterior, middle, and posterior fossae (1–4).

In this study, we evaluated the intracranial vasculature of adult and early postnatal mice using vascular casting combined with ex vivo micro-CT. We studied the intracranial vasculature at E14.5 using gadolinium-enhanced, ex vivo MRI because vascular casting presented significant technical challenges. Our findings of the transition of tentorial venous lakes into a mature and consistent TTVS challenges the previously described hypotheses of human tentorial development (3, 4).

It is currently believed that the tentorial plexus regresses as dural cleavage forms the tentorium and that the superficial and deep venous systems form the adult intracranial venous drainage from the central and lateral head veins (3, 4, 8, 11, 12). However, our finding of a patent tentorial venous drainage system adds a third venous system that necessitates further study. As mentioned, the use of spontaneously aborted human fetuses with possible underlying pathologies could have obscured the understanding of the development of the TTVS in previous developmental studies. In this study, we were able to longitudinally evaluate the development of this system and found that in the postnatal period, when the posterior venous drainage dominates, the tentorial venous lakes connect venous drainage of the anterior, middle, and posterior fossae. Further, we found that these lakes, which we believe transition into the adult TTVS, are continuous with the caudal confluence of sinuses that connects the central head vein, occipital sinus, and marginal sinus.

To our knowledge, this is the first description of the TTVS in mice. Although there have been outstanding studies of intracranial vasculature in mice, tentorial veins have not previously been identified (13). Our results demonstrated that this system is highly conserved between adult mice and humans despite other interspecies variations in brain, skull, and cerebrovascular development. Given this homology, we believe mice can be used as a model to study the development of this venous system and its anatomic, physiologic, and pathologic relationships. For example, idiopathic vascular pathologies of the tentorium, such as tentorial dural arteriovenous fistulas (DAVFs), have been reported to have worse outcomes than DAVFs in other locations (14). We believe that these aberrant connections are likely the embryologic remnants of the tentorial venous plexus that give rise to the TTVS. Based on our findings herein, we believe that the development of these types of pathologies can be studied in mice.

In our evaluation of the embryo, we also found that the developing choroid plexus drains to the tentorial venous plexus. In studies of spontaneously aborted human fetuses, Markowski and Streeter proposed the dynamic reorganization of the early embryologic venous plexuses, including early venous drainage of the fourth ventricle choroid plexus into the tentorial venous plexus (3, 11, 12). It was unclear from those studies if this connection occurred and how the choroid plexus would ultimately drain to the deep venous system. Our finding of choroid plexus venous drainage to the tentorial venous plexus at this stage necessitates further investigation to understand its transition to its adult configuration. Detailed evaluation of these transient embryologic connections can provide insight into their integrations with vasculature, including the formation of the blood–cerebrospinal fluid barrier (15–17).

In this study, we confirmed that the TTVS is present and consistent across mice and humans. We demonstrated that this system is the mature configuration of the persistent tentorial venous plexus. Further, we showed that the embryologic tentorial venous plexus transitions to the adult configuration through tentorial venous lakes in the postnatal period. We also identified the developmental anatomy of venous drainage of the choroid plexus in mice. Our findings in this work add to the understanding of venous development and establish mice as a viable translational model for the study of intracranial vascular pathology.

## Methods

### Vascular perfusion and casting.

We evaluated WT adult (*n* = 4, 1 male and 3 female) and P8 (*n* = 3, 1 male and 2 female) C57BL/6 mice for tentorial venous anatomy of the head via polymer casting and ex vivo micro-CT as previously described (18). Briefly, mice were injected with heparin (1 U/g) and allowed to ambulate prior to euthanasia via CO_2_ narcosis. Bilateral thoracotomy was performed and the abdominal aorta was exposed and cannulated. Sodium nitroprusside (MilliporeSigma) was perfused through the cannulated abdominal aorta to dilate vessels and clear all blood. Microfil polymer (Flowtech Inc.) was then perfused to generate a cast of the murine cerebrovasculature. C57BL/6 mice at E14.5 (*n* = 4, 2 male and 2 female) were evaluated by MRI after immersion in 4% paraformaldehyde for 36 hours, then 0.1% Magnevist/PBS solution for 24 hours. The embryos were placed in 15 mm NMR sample tubes filled with Fluorinert to match the magnetic susceptibility between the embryos and surroundings and positioned in the scanner.

### Image acquisition.

Ex vivo micro-CT of polymer-perfused adult and P8 mice was performed on the Skyscan1172 (Bruker microCT) with a nominal resolution of 13.53 μm, 0.5 mm aluminum filter, and the x-ray source biased at 65 kV and 110 μA. Six projections were averaged together every 0.4° for a 180° scan, each with an exposure time of 1600 ms.

Reconstruction was carried out with a modified Feldkamp_ii_ algorithm using the SkyScan NRecon software accelerated by graphics processing unit. Gaussian smoothing, ring artifact reduction, and beam hardening correction were applied (1, 2).

MRI of gadolinium-immersed mice was performed with a 14T ex vivo tissue scanner. The imaging parameters, including field of view and matrix size, were maintained to achieve a constant isotropic resolution. Images were acquired at 50 μm isotropic resolution echo time of 5 ms, repetition time of 50 ms, and flip angle of 30°.

### Image processing and visualization.

We visualized micro-CT and MRI samples using Horos (Nimble Co, LLC; https://horosproject.org/download-horos/) and Neuroglancer (https://github.com/neurodata/neuroglancer, commit ID aa3670f6480bb6f3525accb5927b306c75a7ac60) for 2D raw data and 3D volume-renders (19). The following common image processing steps were used to generate visualizations from the micro-CT and MRI scans: 1) noise reduction with a Gaussian kernel and 2) creating and 3) applying a transfer function specific to our samples.

### Study approval.

All animals used in this study were treated humanely and in accordance with IACUC/ethics committee approval from the National Institute of Child Health and Human Development, NIH, Bethesda, Maryland, USA.

## Author contributions

PPL analyzed data and drafted and revised the manuscript. Vikram Chandrashekhar generated and analyzed data and drafted and revised the manuscript. Co–first authorship was provided to PPL to Vikram Chandrashekhar in a manner commensurate to the work done by each individual. PPL and Vikram Chandrashekhar contributed equally to this work. AC generated data and revised the manuscript. JPB, Vibhu Chandrashekhar, and JMT analyzed data and revised the manuscript. DRD and JPM generated data and revised the manuscript. JGS, JDH, and ZZ supervised experiments and revised the manuscript. JSR conceived the study, designed the experiments, generated and analyzed data, and drafted and revised the manuscript.

## Supplementary Material

Supplemental data

Supplemental video 1

Supplemental video 2

Supplemental video 3

## Version 1 09/21/2021

In-Press Preview

## Version 2 11/08/2021

Electronic publication

## References

[1] Rosenblum JS (2019). Tentorial venous anatomy: cadaveric and radiographic study with discussion of origin and surgical significance. World Neurosurg.

[2] Rosenblum JS (2020). Tentorial venous anatomy: variation in the healthy population. AJNR Am J Neuroradiol.

[4] Padget DH (1956). The cranial venous system in man in reference to development, adult configuration, and relation to the arteries. Am J Anat.

[5] Kaplan HA (1959). The transcerebral venous system: an anatomical study. AMA Arch Neurol.

[6] Oka K (1985). Microsurgical anatomy of the superficial veins of the cerebrum. Neurosurgery.

[7] Matsushima T (1989). Microsurgical anatomy of the tentorial sinuses. J Neurosurg.

[8] Okudera T (1994). Development of posterior fossa dural sinuses, emissary veins, and jugular bulb: morphological and radiologic study. AJNR Am J Neuroradiol.

[9] Jin S-W (2016). Development and growth of the normal cranial vault: an embryologic review. J Korean Neurosurg Soc.

[10] Maga AM (2016). Postnatal development of the craniofacial skeleton in male C57BL/6J mice. J Am Assoc Lab Anim Sci.

[12] Velut S (1987). [Embryology of the cerebral veins]. Neurochirurgie.

[13] Mancini M (2015). Head and neck veins of the mouse. A magnetic resonance, micro computed tomography and high frequency color doppler ultrasound study. PLoS One.

[14] Li Y Onyx embolization for dural arteriovenous fistulas: a multi-institutional study. J Neurointerv Surg.

[15] Sturrock RR (1979). A morphological study of the development of the mouse choroid plexus. J Anat.

[16] Lun MP (2015). Development and functions of the choroid plexus-cerebrospinal fluid system. Nat Rev Neurosci.

[17] Dani N (2021). A cellular and spatial map of the choroid plexus across brain ventricles and ages. Cell.

[18] Rosenblum JS (2021). Developmental vascular malformations in EPAS1 gain-of-function syndrome. JCI Insight.

[19] Vogelstein JT (2018). A community-developed open-source computational ecosystem for big neuro data. Nat Methods.

