# Supplementary material for: Tentorial venous anatomy of mice and humans
Source: JCI Insight. 2021 Nov 8;6(21):e151222. doi: 10.1172/jci.insight.151222 (PMC8663545; doi:10.1172/jci.insight.151222)
Supplement: Supplemental data [file jciinsight-6-151222-s017.pdf]

Supplementary Materials

Supplementary Figures

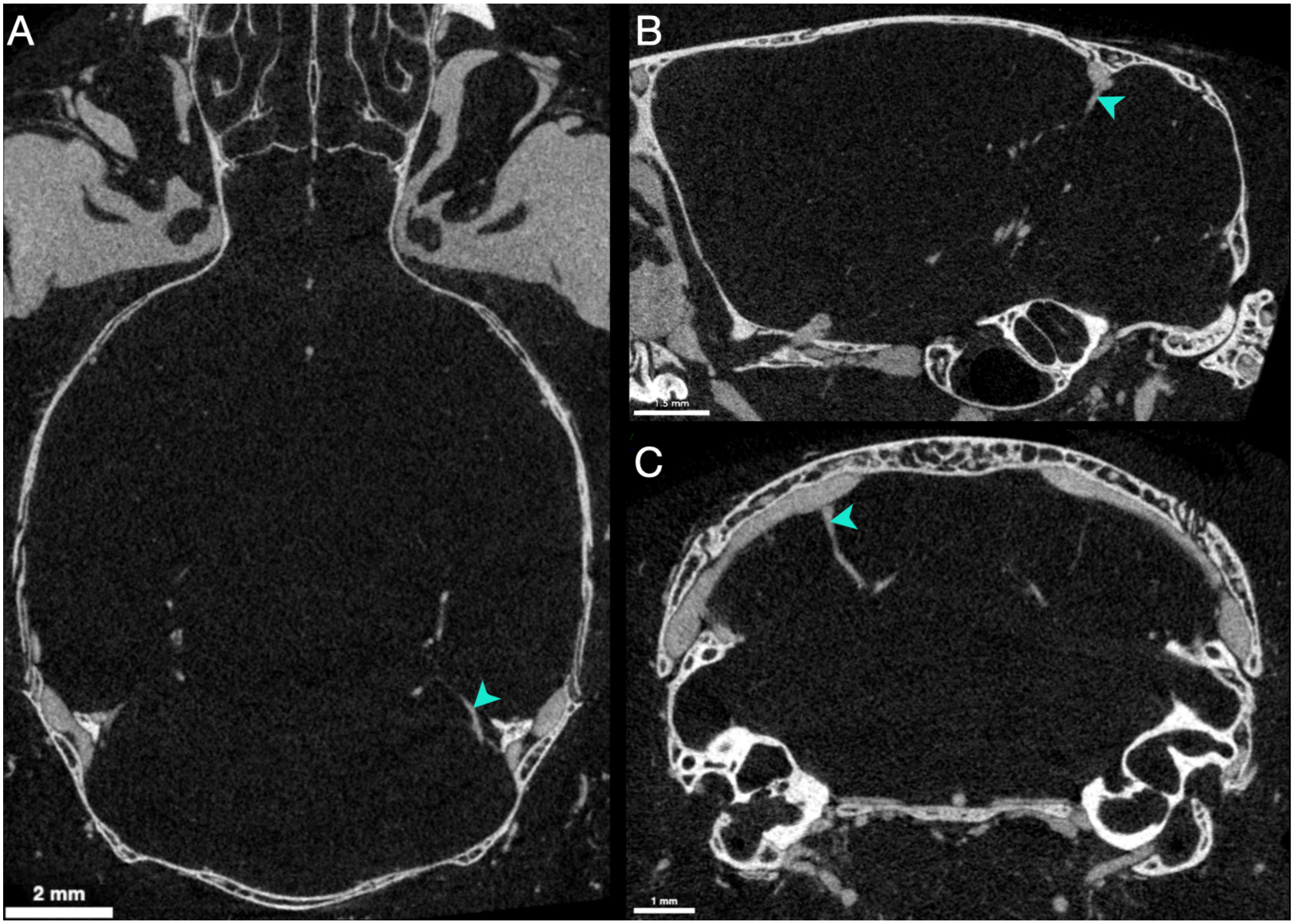

Supplementary Figure 1. **Acquired 2D Micro-CT Images of the Trans-tentorial Venous System (TTVS) in the Adult Mouse.** Raw 2D Micro-CT images of the adult murine head in axial (A), sagittal (B), and coronal (C) views highlighting the TTVS (arrowheads). (n=4, 1 male and 3 female).

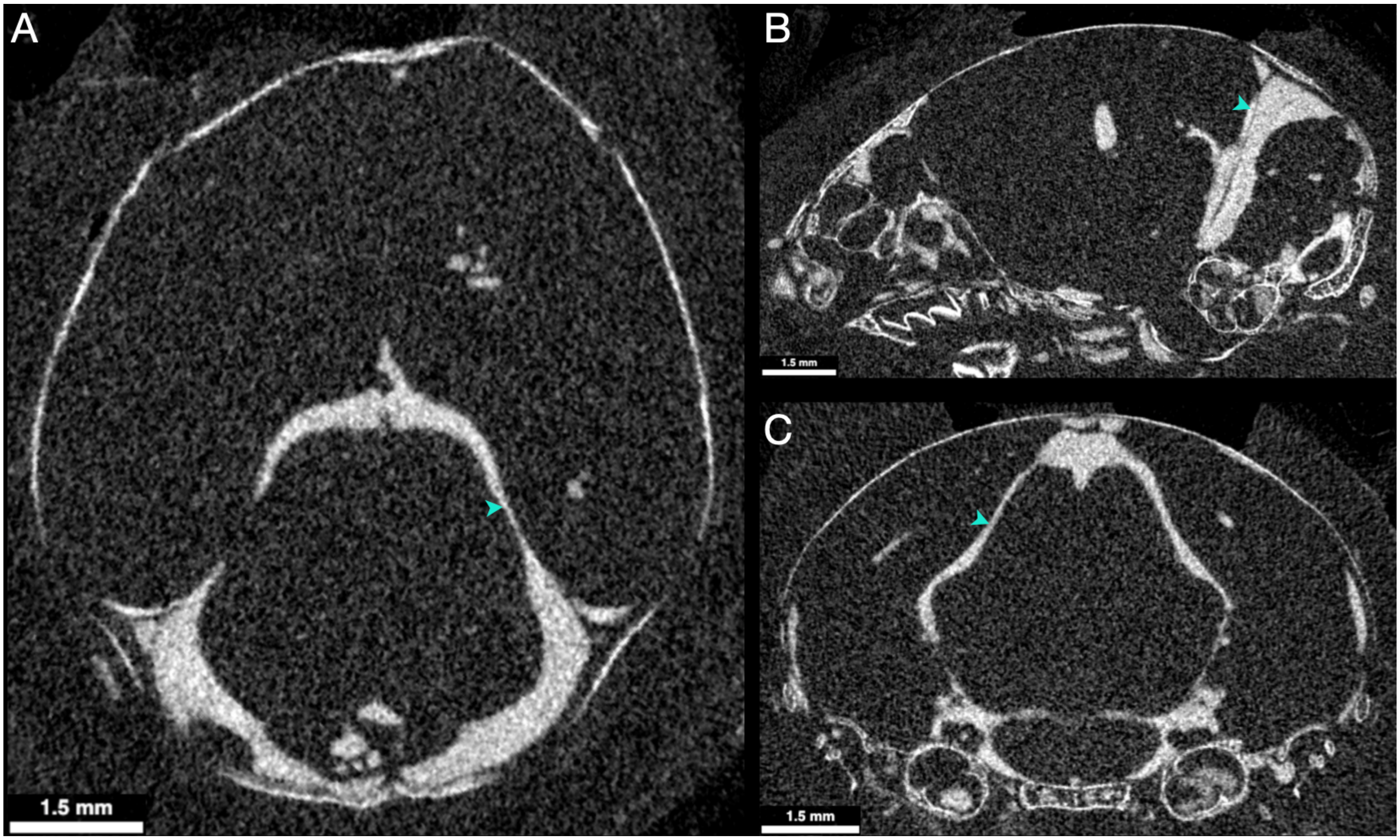

Supplementary Figure 2. **Acquired 2D Micro-CT Images of the Trans-tentorial Venous System (TTVS) in the Post-Natal Day 8 Mouse Head.** Raw 2D Micro-CT images of the post-natal mouse head in axial (A), sagittal (B), and coronal (C) views highlighting the tentorial venous lakes (arrowheads). (n=3, 1 male and 2 female).

Supplementary Videos

Supplementary Video 1. **3D Visualization of Micro-CT Images of the Trans-tentorial Venous System (TTVS) in the Adult Mouse Head.** Flythrough of the adult mouse head in multiple views. The TTVS is annotated in a coronal and parasagittal view. (n=4, 1 male and 3 female). 3D indicates three-dimensional; Micro-CT, micro computed tomography. BA, basilar artery; CS, cavernous sinus; ITV, intermediate tentorial vein; LTS, lateral tentorial sinus; LTV, lateral tentorial vein; MTV, medial tentorial vein; SPS, superior petrosal sinus; StS, straight sinus; TS, transverse sinus; HpV, hippocampal veins; MR, magnetic resonance; TS-SS J, transverse sinus-sigmoid sinus junction.

Supplementary Video 2. **2D and 3D Visualization of MR Images of the Tentorial Venous Plexus in the E14.5 Mouse.** Flythroughs of the mouse embryo in multiple views. The embryologic TTVS is annotated in a coronal and parasagittal view. (n=4, 2 male and 2 female). 2D indicates two-dimensional; 3D, three-dimensional; CP, choroid plexus; TVP, tentorial venous plexus; PVP, posterior venous plexus;

Supplementary Video 3. **3D Visualization of Micro-CT Images of the Tentorial Venous Lakes (TVL) in the Post-Natal Mouse Head at Day 8.** Flythrough of the post-natal mouse head in multiple views. The TVLs and associated sinuses are annotated in a coronal and parasagittal view. (n=3, 1 male and 2 female). 3D indicates three-dimensional; CoS, confluence of sinuses; EJV, external jugular vein; FV, facial vein; Micro-CT, micro-computed tomography; OS, occipital sinus; TVL, tentorial venous lake
